# Uteroplacental–Cerebral Ratio: A Doppler Parameter for Prognostic Prediction of Late-Onset Fetal Growth Restriction: Single Center Prospective Cohort Study

**DOI:** 10.3390/jcm12010275

**Published:** 2022-12-29

**Authors:** Ziling Yang, Wenjie Lv, Baojing Zhao, Jie Yao, Yuanyuan Yang, Zongzhi Yin

**Affiliations:** 1 Department of Obstetrics and Gynecology, The First Affiliated Hospital of Anhui Medical University, Hefei 230022, China; 2 NHC Key Laboratory of the Study of Abnormal Gametes and the Reproductive Tract, Anhui Medical University, Hefei 230022, China

**Keywords:** fetal growth restriction, cerebroplacental ratio, uterine artery, umbilical artery, middle cerebral artery, pulsatility index

## Abstract

Purpose: This study aimed to elucidate the accuracy of Doppler parameters in predicting the prognosis of late-onset fetal growth restriction (FGR). Methods: This was a prospective study of 114 pregnancies. Doppler parameters, including the cerebroplacental ratio and pulsatility index (PI) in the middle cerebral, umbilical, uterine artery, were recorded. The new uteroplacental–cerebro ratio (UPCR) was constructed as the ratio of (umbilical artery + mean of the left and right uterine artery) to middle cerebral artery PI. Logistic regression analyses and receiver operating characteristic curves were performed. Results: Adverse outcomes occurred in 37 (32%) neonates. The z values of the middle cerebral artery PI and cerebroplacental ratio were lower (*p* < 0.001), while the z values of the umbilical artery PI, mean uterine artery PI, and UPCR (*p* < 0.001) were higher in late-onset FGR in those with compared to those without adverse outcomes. Multivariate logistic regression revealed that only UPCR was independently associated with adverse outcomes (*p* < 0.001). For predicting the prognosis of late-onset FGR, UPCR showed a fair degree of accuracy (area under the curve [AUC], 0.824). Conclusion: The new UPCR, reflecting the impact of placental impedance from both fetal and maternal sides on fetal well-being, improves the accuracy of prognostic prediction for late-onset FGR.

## 1. Introduction

Fetal growth restriction (FGR) occurs when the fetus does not reach its intrauterine potential for growth and development. Late-onset FGR is FGR diagnosed at >32 weeks [1,2,3]. The incidence of late-onset FGR is probably 5–10% and it is one of the leading causes of perinatal mortality and morbidity [1]. In the absence of any effective treatment for fetal growth restriction, the mainstay of management is close surveillance and timely delivery [4,5]. Thus, the identification of fetuses at high risk of perinatal compromise is crucial to maximize the chances of good outcome of pregnancies affected by late-onset FGR.

Placentas from late-onset FGR fetuses present histological signs of placental underperfusion, such as vascular occlusion and villous hypoplasia [6]. Doppler manifestations of increased placental vascular resistance are frequently associated with adverse pregnancy outcomes of fetal growth restriction [7,8,9,10]. Blood flow of the umbilical artery (UA) represents the degree of perfusion to the fetal–placental unit [5,11,12]. Unfortunately, evidence has shown that umbilical artery Doppler does not reliably predict adverse perinatal outcomes of late-onset FGR [11,12] because the UA Doppler becomes abnormal only if an extensive part of the placenta is involved. It has been shown that in near-term FGR fetuses, middle cerebral artery (MCA) Doppler can be used to predict adverse outcomes with an acceptable specificity, but with a low sensitivity for clinical applicability [10,11]. Abnormal uterine artery (UtA) Doppler, which represents maternally under-perfused placentas, is associated with inadequate trophoblast invasion. However, it is questionable whether the uterine artery should be used as a predictive index because of its varied results in predictive accuracy [13,14]. Notably, the cerebroplacental ratio (CRP) has been demonstrated to be more sensitive to hypoxia, which improves the sensitivity of UA and MCA alone [15,16,17]. However, its predictive value for adverse outcomes in the third trimester remains uncertain [18,19]. Thus, the need for more accurate Doppler predictors of adverse outcomes in pregnancies affected by late-onset FGR should be strongly considered.

The present study proposes a new uteroplacental–cerebral ratio (UPCR), an index reflecting the impact of combined placental vascular impedance from the maternal and fetal sides on fetal well-being. The purpose of this study was to investigate the diagnostic performance of commonly used Doppler ultrasound and UPCR on adverse pregnancy outcomes in late-onset FGR.

## 2. Materials and Methods

### 2.1. Study Population

Between August 2020 and December 2021, a prospective cohort of consecutive singleton pregnancies with suspected FGR was recruited among patients attending the outpatient unit of the Department of Obstetrics and Gynecology, The First Affiliated Hospital of Anhui Medical University, for routine third-trimester screening. Fetuses diagnosed with late-onset FGR were defined according to the consensus [20], including abdominal circumference/estimated fetal weight (AC/EFW) < 3rd percentile or at least two out of the three following considerations: (a) AC/EFW < 10th percentile, (b) AC/EFW crossing percentiles >2 quartiles on growth centiles, and (c) CPR < 5th percentile or PI of UA > 95th percentile. Estimated fetal weights were calculated from measurements of the head circumference (HC), biparietal diameter (BPD), abdominal circumference (AC), and femur length (FL) using the customized NICHD fetal growth curves (Asian) [21]. Gestational age was determined by the crown–rump length at 11–13 weeks. The gestational age at the time of the Doppler ultrasound study ranged from 32 + 0 to 37 + 0 weeks, and all fetuses were delivered at ≥32 weeks of gestation. This study protocol was approved by the local institutional ethical committee (PJ2010-12-44). Each pregnant woman who participated in the study signed a written informed consent form.

### 2.2. Doppler Ultrasound Studies

All pregnancies with suspected FGR by ultrasound examination in the outpatient obstetric examination will be recruited. Patients will receive ultrasound examination by one of two senior sonographers again, and those who meet the diagnosis of late-onset FGR will be enrolled in the study. The Cohen’s kappa coefficient of the two sonographers was 0.918 (*p* < 0.001), indicating a high degree of concordance in their measurements. Ultrasounds were performed with a 3.5–5.0-MHz transducer and Voluson E8 machines (GE Healthcare, USA). Transabdominal color Doppler ultrasound was used to measure the Doppler velocity waveforms from the umbilical artery, middle cerebral artery, and uterine artery [12,13,14]. Briefly, the umbilical artery was recorded from a free-floating cord loop [12,13]. The middle cerebral artery was measured at an angle of nearly 0° from its origin to the Willis loop [12]. To record the uterine artery Doppler, the transducer was placed over the iliac fossa, and pulsed Doppler was applied 1 cm medial to the crossover point of the uterine and iliac vessels [14]. The pulsatility index (PI) in MCA, UA, and UtA was recorded. The CPR value was calculated as MCA-PI/UA-PI. A new index, the uteroplacental cerebral ratio (UPCR), was constructed as (UA-PI + mean of the left and right UtA-PI)/MCA-PI. It is a Doppler index which was used to evaluate the impact of placental vascular impedance from the fetal and maternal sides on fetal well-being. As Doppler indices vary with gestational age, the values were converted to gestation-specific MoMs. Measurements were taken in the absence of fetal breathing or movement. All PI values were measured in triplicate and the mean was used for statistical analysis.

### 2.3. Clinical Management and Outcome Measures

Pregnancies recruited in the study were managed by obstetricians who were not involved in the late-onset FGR diagnostic process. They were not aware of the ultrasound Doppler data other than UA-PI. Pregnant women were monitored weekly or twice a week for clinical assessment, amniotic fluid volume and hemodynamic changes according to local protocols. Fetal growth was reassessed every two weeks. The decision regarding the time and mode of delivery was made based on the gestational age, nonreassuring fetal testing, ultrasound estimation of the amniotic fluid index, umbilical artery Doppler, fetal growth, and/or maternal signs of severe preeclampsia. Delivery was indicated in the presence of (a) worsening maternal or fetal conditions as determined by the managing physician, (b) nonreassuring fetal testing, (c) absent or reversed end-diastolic blood flow in the umbilical artery, and (d) gestational age beyond 39 weeks of gestation. The primary outcome of the present study was defined as the occurrence of composite adverse perinatal outcomes, which included at least one of the following complications: stillbirth, emergency cesarean section (CS) for fetal distress, 5-min Apgar score < 7, umbilical artery pH < 7.10, and neonatal admission to the special care unit. Newborns need to be transferred to the neonatal special care unit when the following conditions occur: the need for resuscitation at birth, need for respiratory support at birth, pathological jaundice, neonatal hypoglycemia, and aspiration pneumonia.

### 2.4. Statistical Analysis

Categorical variables were compared using χ^2^ analysis (Exact) or Fisher’s exact test. Continuous variables are presented as the means ± standard deviations (SDs) and were compared using Student’s *t*-test and Mann-Whitney U test. Multivariate logistic regression analyses were performed with the backward stepwise (LR) method to investigate the association between the different Doppler parameters and adverse pregnancy outcomes in late-onset FGR. The adjusted odds ratios (aORs) and 95% confidence intervals (95% CIs) were determined. Receiver-operating characteristic (ROC) curves were constructed for each predictor to elucidate the diagnostic performance in predicting adverse perinatal outcomes. The Youden index was used to determine the optimal cut-off value. The sensitivity, specificity, positive predictive value, and negative predictive value with 95% CIs were calculated and compared. A two-sided *p* value of <0.05 was considered statistically significant. MedCalc (version 19.0.4) and SPSS software (version 25.0, Chicago, IL, USA) were used for statistical analysis and graph construction.

## 3. Results

**Figure 1:**
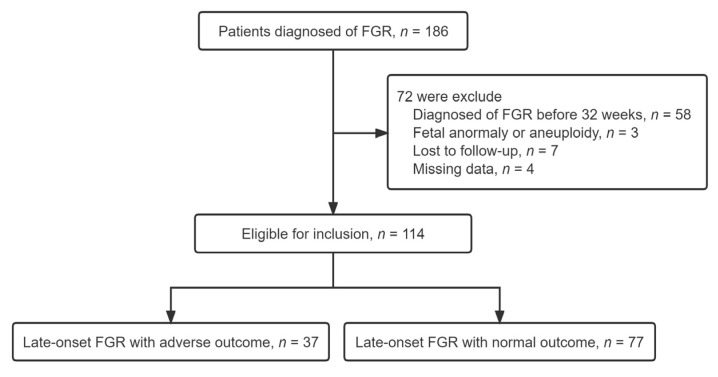
Process of pregnant patient inclusion beginning from the initial search to final inclusion. A total of 186 participants were excluded from the analyses because they were subsequently lost to follow-up, missing data, diagnosed with early-FGR, congenital malformations or aneuploidy. There were 114 eligible women enrolled. Thirty-seven pregnant women with late-onset FGR had adverse perinatal outcomes and were assigned to the study group; the remaining 77 pregnant women with late-onset FGR had normal perinatal outcomes and were assigned to the control group.

A total of 186 pregnant patients with suspected FGR were recruited. 72 patients were excluded due to diagnosing of early FGR (*n* = 58), losing to follow-up (*n* = 7), missing data (*n* = 4), fetal congenital malformations or aneuploidy (*n* = 3), leaving 114 pregnancies available for analysis (Figure 1). The composite adverse outcome occurred in 37 (32%) infants and the prevalence was detailed in the following table. Emergency CS for fetal distress, umbilical artery pH < 7.10, Apgar < 7 at 5-min and neonatal admission to special care were recorded in 16 (10.52%), 4 (3.50%), 14 (12.28%), and 27 (23.68%) cases, respectively, while no case of stillbirth occurred in the study.

The characteristics of the study population are reported in Table 1. Compared with the pregnant women without adverse perinatal outcomes, the mean birthweight (2059.72 ± 232.42 vs. 2358.70 ± 296.13, *p* < 0.001), birthweight percentile (4.17 ± 2.26 vs. 5.76 ± 2.89, *p* = 0.004) and gestational age at birth (35.52 ± 1.5 vs. 37.39 ± 1.75, *p* < 0.001) were lower in the pregnancies who experience adverse perinatal outcomes, while there was no difference regarding maternal age, BMI, gestational age at detection, nulliparity, previous FGR, previous stillbirth, gestational diabetes, hypertensive diseases, or liver disease disorders between the two study groups (*p* > 0.05).

In Table 2, when exploring the differences between the Doppler parameters, the MoMs of MCA PI (0.69 ± 0.14 vs. 0.84 ± 0.19, *p* < 0.001) and CPR (0.49 ± 0.20 vs. 0.69 ± 0.24, *p* < 0.001) were lower, while the UA PI MoMs (1.48 ± 0.48 vs. 1.21 ± 0.32, *p* < 0.001), mean uterine artery PI MoMs (2.23 ± 0.53 vs. 1.59 ± 0.70, *p* < 0.001) and UPCR MoMs (2.74 ± 0.76 vs. 1.74 ± 0.73, *p* < 0.001) were higher in late-onset FGR pregnancies with adverse perinatal outcomes than in the control group (*p* < 0.001).

Multivariate logistic regression analysis was used to combine z values of MCA-PI, UA-PI, mean UtA-PI, CPR, UPCR, birthweight, preeclampsia, and gestational age in stepwise regression analysis to predict adverse outcomes of late-onset FGR. The results showed that only UPCR, with higher odds, was independently associated with adverse pregnancy outcomes of late-onset FGR (aOR = 4.74, 95%CI = 2.26–9.93, *p* < 0.001, Table 3). The ROC curve analysis of the diagnostic performance of Doppler parameters for prognostic prediction of late-onset FGR is shown in Table 4. The AUC of UPCR (0.824, 95% CI 0.739–0.891), was significantly higher than MCA (AUC:0.705, 95% CI 0.610–0.789; *p* = 0.025 vs. UPCR) and UA PI (AUC: 0.710, 95% CI 0.615–0.794; *p* = 0.038 vs. UPCR), but was slightly higher than that of CPR (AUC: 0.765, 95% CI 0.673–0.841; *p* = 0.245 vs. UPCR), and UtA PI (AUC:0.753, 95% CI 0.661–0.831; *p* = 0.107 vs. UPCR). However, UPCR had a higher sensitivity (80.5%) and a comparable specificity (72.9%) for predicting adverse outcomes in late-onset FGR than CPR and mean UtA PI, which indicated that UPCR may be a better prediction index for prognostic prediction of late-onset FGR (Table 4).

## 4. Discussion

This study showed that about 32% of late-onset FGR occurred with adverse perinatal outcomes, which is consistent with previous literature, [11,14], confirming a high rate of perinatal damage in pregnancies affected by FGR. Until now, no treatment has been demonstrated to benefit late-onset FGR. Therefore, the timely identification of the fetus at greatest risk for adverse outcome is crucial for the preventive treatment of late-onset FGR [4,5]. This study proposes a new ultrasound Doppler parameter, UPCR, which reflects the influence of vascular impedance from both sides of the placenta on the fetus. Compared with late-onset FGR pregnancies of normal outcome, cases with adverse perinatal outcomes had higher UPCR, UA-PI, and UtA-PI but lower CRP and MCA-PI. After adjustment for confounders, only UPCR was independently associated with adverse pregnancy outcomes of late-onset FGR. When exploring the diagnostic performance of Doppler in predicting adverse outcomes, UPCR showed a fair accuracy, which was better than CPR, UA-PI, UtA-PI, and MCA-PI. Among the Doppler indices tested, abnormal UPCR showed a higher sensitivity for predicting adverse pregnancy outcomes in late-onset FGR compared with conventional MCA, UA, and UtA PIs, separately.

Clinical evolution of late-onset FGR is associated with placental insufficiency, which leads to reduced oxygen as well as a slight increase of placental resistance [6]. Umbilical artery Doppler is the reflection of placental resistance from the fetal placental side [5,8,9]. Late onset FGR is usually caused by milder placental disease that results in a subtle but chronic state of hypoxia and malnutrition that is usually not well reflected by changes in classical UA Doppler [8,9]. The CPR, which combines the pulsatility index of the MCA and UA, has been demonstrated to be more sensitive to hypoxia than its individual components [15]. However, the largest prospective series on unselected pregnancies failed to find the predictive value of CPR adverse outcomes when performed at 30–34 or 35–37 weeks [16,17]. A previous study showed that CPR had a moderate accuracy with an AUC of 0.615 for predicting adverse outcomes of late-onset FGR [11]. Therefore, the effectiveness of a strategy based on the CPR assessment in late-onset FGR remains to be proven. Uterine artery Doppler, which represents the maternal side of placental resistance, was doubtful to be a surveillance tool of late-onset FGR [13,14]. In a study by Obican, the mean UtA PI had a low diagnostic performance for predicting composite adverse outcomes of neonatal SGA with a low sensitivity of 41% and a moderate specificity of 70% [14]. In addition, UtA-PI proved to have no significant correlation with adverse neonatal outcomes of late-onset FGR [18].

In order to improve the accuracy rate, combinations of the associated Doppler parameters were used to further improve the detection of FGR. Gudmundsson, et al. put forward a new placental pulsatility index (PPI, an average of UA PI and UtA PI) which has a high sensitivity for the prediction of adverse pregnancy outcomes in suspected FGR [22]. MacDonald et al. put forward a new cerebral-placental-uterine ratio (CPUR; CPR/UtA PI) as a novel predictor of late-onset FGR with superior sensitivity [23]. In this study, we innovatively propose uteroplacental–cerebral ratio (UPCR), a new Doppler index reflecting the uteroplacental insufficiency. The UPCR combines Doppler parameters that each represent unique biological manifestations of placental insufficiency: raised UA PI indicates increased placental resistance to fetal blood [5,8]; increased UtA PI may indicate poor perfusion of the uterine artery to the placenta [13] and decreased MCA PI is an adaptation of the fetus to hypoxia [9]. The result showed that UPCR was independently associated with adverse pregnancy outcomes of late-onset FGR. Abnormal UPCR had a higher sensitivity, as well as a considerable specificity, than the conventional Doppler parameters, indicating excellent diagnostic performance for the prediction of adverse pregnancy outcomes in late-onset FGR.

In this study, we compared the predictive value of commonly used ultrasound Doppler parameters for adverse pregnancy outcomes in late-onset FGR. The strengths of our study include its original prospective design and the ability to access the third-trimester Doppler parameters, which were less frequently evaluated before. The main limitations of this study include a relatively small sample size, for which neonatal outcome had to be evaluated as a composite outcome. As UPCR values vary with gestational weeks, trying to obtain an exact cut-off value is difficult. And it was not appropriate to use the cut-off values based on previous studies for statistical comparison because the target population of the study was different. Therefore, we applied the Youden index to determine the optimal cut-off value. The values were only used for statistical comparison but not suitable for clinical application, so they were not presented in this study. Besides, the present study did not take into account any serial change in the Doppler index as each pregnancy progressed from diagnosis to delivery.

In summary, we propose a new Doppler index (UPCR), reflecting the impact of placental vascular impedance from the fetal and maternal sides of the placenta on fetal well-being. The UPCR is independently correlated with adverse perinatal outcomes of late-onset FGR, with higher diagnostic accuracy, sensitivity, and considerable specificity than the conventional Doppler parameters, which may be a better predictor for the prognosis of late-onset FGR. It might, therefore, improve the detection of late-onset FGR health status by Doppler ultrasound and assist in planning surveillance of pregnant women with late-onset FGR. However, this conclusion needs to be further verified in a large-sample prospective study.

## Figures and Tables

**Table 1:** Maternal characteristics of the enrolled pregnancies with late-onset FGR.

Characteristics	Normal Outcome (*n* = 77)	Adverse Outcome (*n* = 37)	*p*
Age (years)	30.1 ± 4.6	29.8 ± 5.7	0.804 ^a^
BMI (kg/m^2^)	28.4 ± 4.5	29.3 ± 5.6	0.179 ^a^
Obstetric history (*n*, %)			
Nulliparous	48 (62.3)	19 (51.3)	0.265 ^b^
Previous FGR	3 (3.8)	4 (10.8)	0.150 ^c^
Previous stillbirth	0(0.0)	1(2.7)	0.325 ^c^
Pregnancy complication (*n*, %)			
Gestational diabetes	6 (7.9)	3 (8.1)	0.953 ^c^
Liver diseases	5 (6.9)	2 (5.4)	0.821 ^c^
Hypertensive diseases	46 (59.7)	26 (70.2)	0.275 ^b^
No comorbidity	12 (15.5)	4 (10.8)	0.576 ^b^
Gestational age at detection (weeks)	34.0 ± 1.4	33.6 ± 1.5	0.108 ^a^
Mean gestational age at birth (weeks)	37.3 ± 1.7	35.5 ± 1.5	<0.001 ^a^
Birthweight (g)	2358.7 ± 296.1	2059.7 ± 232.4	<0.001 ^a^
Birthweight percentile (%)	5.7 ± 2.8	4.1 ± 2.2	0.004 ^a^

Values are expressed as mean ± SD (range) or number (percentage). Abbreviations: BMI, body mass index; FGR, fetal growth restriction. ^a^ Independent samples *t*-test (Bootstrap); ^b^ Pearson χ^2^ test (Exact); ^c^ Fisher’s exact test (Exact).

**Table 2:** Ultrasound Doppler indices of the pregnant women enrolled.

Variables	Normal Outcome (*n* = 77)	Adverse Outcome (*n* = 37)	*p*
EFW < 3 centile	20 (25.97)	18 (48.46)	0.016 ^a^
MCA PI MoM	0.84 ± 0.19	0.69 ± 0.14	<0.001 ^b^
UA PI MoM	1.21 ± 0.32	1.48 ± 0.48	<0.001 ^b^
Mean UtA PI MoM	1.59 ± 0.70	2.23 ± 0.53	<0.001 ^b^
CPR MoM	0.69 ± 0.24	0.49 ± 0.20	<0.001 ^b^
UPCR MoM	1.74 ± 0.73	2.74 ± 0.76	<0.001 ^b^

Values are expressed as mean ± SD (range) or number (percentage). Abbreviations: EFW, estimated fetal weight; UA, umbilical artery; MCA, middle cerebral artery; UtA, uterine artery; CPR, cerebroplacental ratio; UPCR, uterine-placental-cerebro ratio; PI, pulsatility index. ^a^ Pearson χ^2^ test (Exact); ^b^ Mann-Whitney U test.

**Table 3:** Multivariate logistic regression analysis of the association of Doppler indices with adverse perinatal outcomes of late-onset FGR.

Variables	B	SE	Adjusted OR	95%CI	*p*
MCA PI MoM	−1.243	1.621	0.288	0.01–6.91	0.443
UA PI MoM	0.385	0.751	1.469	0.33–6.40	0.608
Mean UtA PI MoM	0.286	0.555	1.330	0.44–3.94	0.607
CPR MoM	−1.580	1.497	0.206	0.01–3.87	0.291
UPCR MoM	1.557	0.377	4.744	2.26–9.93	<0.001
Gestational age at delivery	−0.285	0.186	0.752	0.52–1.10	0.125
Birthweight percentile (%)	−0.095	0.100	0.909	0.74–1.10	0.345
Pre-eclampsia	0.650	0.554	1.916	0.64–5.67	0.240

Abbreviations: B, regression coefficient; SE, standard error; OR, odds ratios; 95% CI, 95% confidence interval.

**Table 4:** Predictive performance of Doppler parameters for adverse perinatal outcome of late-onset FGR.

Variables	AUC (95% CI)	Sensitivity (95% CI)%	Specificity (95% CI)%	PPV (95% CI)%	NPV (95% CI)%	*p*
MCA PI MoM	0.705 (0.610–0.789)	62.0 (50.6–73.1)	81.0 (64.8–92.0)	87.3 (75.4–94.8)	50.8 (37.5–64.1)	<0.001
UA PI MoM	0.710 (0.615–0.794)	59.7 (47.9–70.8)	70.2 (53.0–84.1)	80.7 (68.1–90.0)	45.6 (32.4–59.3)	<0.001
Mean UtA PI MoM	0.753 (0.661–0.831)	62.5 (50.3–73.6)	86.4 (71.2–95.5)	90.0 (78.0–96.7)	54.2 (40.6–67.4)	<0.001
CPR MoM	0.765 (0.673–0.841)	74.0 (62.8–83.4)	62.1 (44.8–77.5)	80.3 (69.1–88.8)	53.5 (37.7–68.8)	<0.001
UPCR MoM	0.824 (0.739–0.891)	80.5 (64.0–91.8)	72.9 (61.4–82.6)	88.5 (77.7–95.3)	59.2 (44.2–73.0)	<0.001

Abbreviations: AUC, area under the curve; 95% CI, 95% confidence interval; PPV, positive predictive value; NPV, Negative predictive value.

## Data Availability

The datasets used and/or analyzed during the current study are available from the corresponding author’s e-mail yinzongzhi@ahmu.edu.cn on reasonable request.
